# Stress distribution and displacement of three different types of micro-implant assisted rapid maxillary expansion (MARME): a three-dimensional finite element study

**DOI:** 10.1186/s40510-021-00357-5

**Published:** 2021-06-21

**Authors:** C. B. André, J. Rino-Neto, W. Iared, B. P. M. Pasqua, F. D. Nascimento

**Affiliations:** 1 Technology Research Center, University of Mogi das Cruzes, Dr. Cândido Xavier de Almeida Souza Avenue, 200, Mogi das Cruzes 08780-91 São Paulo, Brazil; 2 Department for Orthodontics, University of São Paulo, Professor Lineu Prestes Avenue, 2227, São Paulo, SP 05508-000 Brazil; 3 Private Practice, Palestina Street,51, Jardim Europa, Vargem Grande Paulista, São Paulo, 06730-000 Brazil; 4 School of Orthodontics, University of São Paulo, Professor Lineu Prestes Avenue, 2227, São Paulo, 05508-000 Brazil

## Abstract

**Abstract:**

**Background/objective:**

Until 2010, adults underwent surgical treatment for maxillary expansion; however, with the advent of micro-implant-assisted rapid maxillary expansion (MARME), the availability of less invasive treatment options has increased. Nevertheless, individuals with severe transverse maxillary deficiency do not benefit from this therapy. This has aroused interest in creating a new device that allows the benefit of maxillary expansion for these individuals. The aim of this study was to evaluate the efficacy of three MARME models according to tension points, force distribution, and areas of concentration in the craniofacial complex when transverse forces are applied using finite element analysis.

**Materials and methods:**

Digital modeling of the three MARME models was performed. Model A comprised five components: one body screw expander and four adjustable arms with rings for mini-implant insertion. These arms have an individualized height adjustment that allows MARME positioning according to the patient’s palatal anatomy, thereby preventing body screw expander collision with the lateral mucosa in severe cases of maxillary deficiency. Model B was a maxillary expander with screw rings joined to the body, and model C was similar to model B, except that model C had open rings for the insertion of the mini-implants. Through the MEF (Ansys software), the stresses, distribution, and area of concentration of the stresses were evaluated when transverse forces of 7.85 N were applied.

**Results:**

The three models maintained the following pattern: model C presented weak stress peaks with limited distribution and lower concentration area, model B obtained median stress peaks with better distribution when compared to that of model C, and model A showed better stress distribution and larger concentration area. In model A, tensions were located in the lateral lamina of the pterygoid process, which is an important site for maxillary expansion. The limitation of the present study was that it did not include the periodontal tissues and muscles in the finite element method evaluation.

**Conclusions:**

Model A showed the best stress distribution conditions. In cases of severe atresia, model A seems to be an excellent option.

**Supplementary Information:**

The online version contains supplementary material available at 10.1186/s40510-021-00357-5.

## Background

Transverse maxillary deficiency affects 13.3% to 18% of individuals with deciduous and mixed dentition [1, 2], and its prevalence is almost 10% in adults [3]. This skeletal change may cause alterations in facial morpho-physiology. The treatment of transverse maxillary deficiency consists of opening the midpalatal suture and separating the hemi-maxillae by rapid maxillary expansion (RME). This procedure was first described by Angell in 1860 [4] and later, Haas [5] initiated studies to assess the effects of this therapy. RME is used successfully in growing patients [6] when the midpalatal suture is not yet fully mature. However, in patients treated after the growth peak, skeletal maturity is shown with resistance zones in the midpalatal suture, limiting the success of RME [6]. Thus, for many specialists, treatment of transverse maxillary deficiency involves surgical procedures, which are invasive and costly [7]. Commonly, most patients refuse invasive procedures, such as surgically assisted rapid maxillary expansion (SARME). Additionally, patients’ financial condition should also be considered before selecting the best treatment option.

In 2010, the first case of RME using mini-implants was reported [8]. This technique is called mini-implant-assisted rapid maxillary expansion (MARME) and is currently used as another option besides SARME8. Recently, Moon et al. developed a device with rings to determine the insertion site of mini-implants [9]. Other models were developed following the same concept introduced by Moon et al., with some variations. These devices have proven to be effective in most cases [9–10]. However, for patients with severe transverse maxillary deficiency, the depth of the palate increases. Furthermore, the size of the screw body expander of these devices can cause tissue damage in the lateral mucosa of the palate in patients with severe maxillary deficiency [11]. Additionally, this technique still presents limitations in cases of asymmetry. Some new MARME models with extension arms allow positioning of the body screw expander without colliding with the lateral palatal mucosa; therefore, they are designed to be used even in severe cases of maxillary deficiency and asymmetry.

The finite element method (FEM) has been applied to evaluate force systems of orthodontic appliances, which enhances results and reduces possible side effects by avoiding clinical tests in humans [12, 13, 14]. FEM can elaborate the uses of MARME by evaluating the stresses and tension of the appliance, since different structures of the craniofacial complex can be modeled and evaluated for the impact analysis of any type of applied force [14, 15, 16].

Since the new models promise to be effective, even in severe cases of transverse maxillary deficiency, it is fundamental to certify their benefits and efficiency in relation to craniofacial tensions and their distribution. As such, the aim of this study was to evaluate and compare the efficacy of three different MARME appliances according to tension points, force distribution, and areas of concentration in the craniofacial complex by FEM.

## Methods

**Fig. 1:**
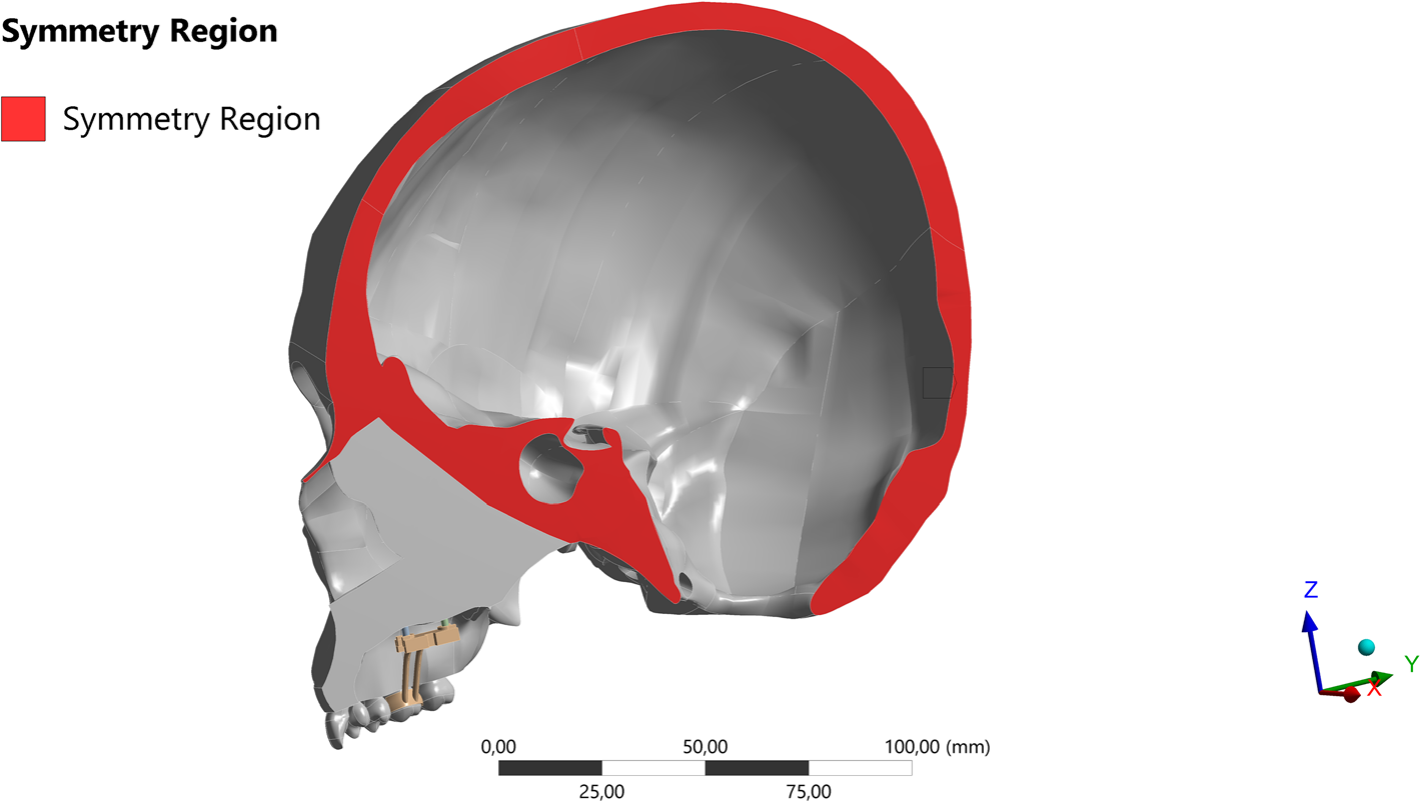
Three-dimensional model of a half human skull obtained from a DICOM file of an adult individual

Since this was an in silico study, where all hypotheses were tested digitally, no ethical approval was needed. A finite element model corresponding to half of the skull of an adult human, obtained from a multi-slice computed tomography (Digital Imaging and Communications in Medicine), was generated (Fig. 1) in Renato Archer Technology Information Center (Campinas, Brazil). The model was duplicated with a symmetrical cranium to exactly comprehend the dissipation of forces. This model was created using the software GR model Light-Speed 16 Pro. The elastic properties of the skull (Table 1) were based on previous studies [12, 17].

**Table 1:** Elastic properties of the skull model

Material	Modulus of elasticity	Poisson’s ratio
Cancellous bone	13.700	0.3
Compact bone	7.930	0.3
Tooth	20.000	0.3

**Fig. 2:**
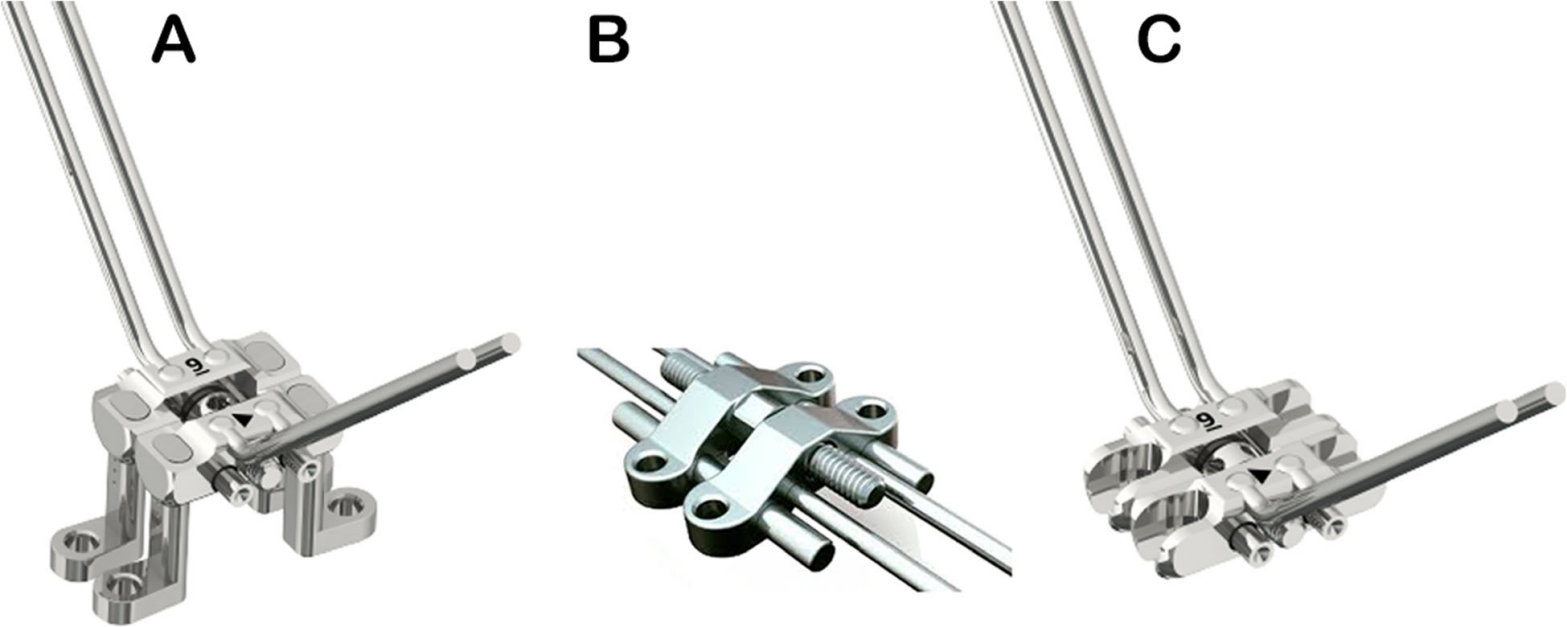
Original design of MARME models. Source: Biomaterial Korea®, Seoul, South Korea and Peclab®, Belo Horizonte, Brazil

**Fig. 3:**
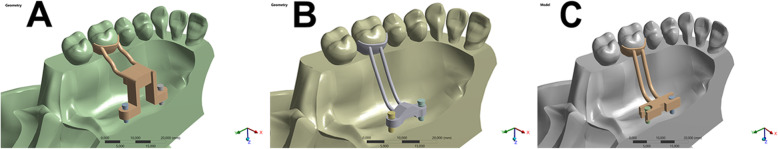
Design of the MARME models

**Fig. 4:**
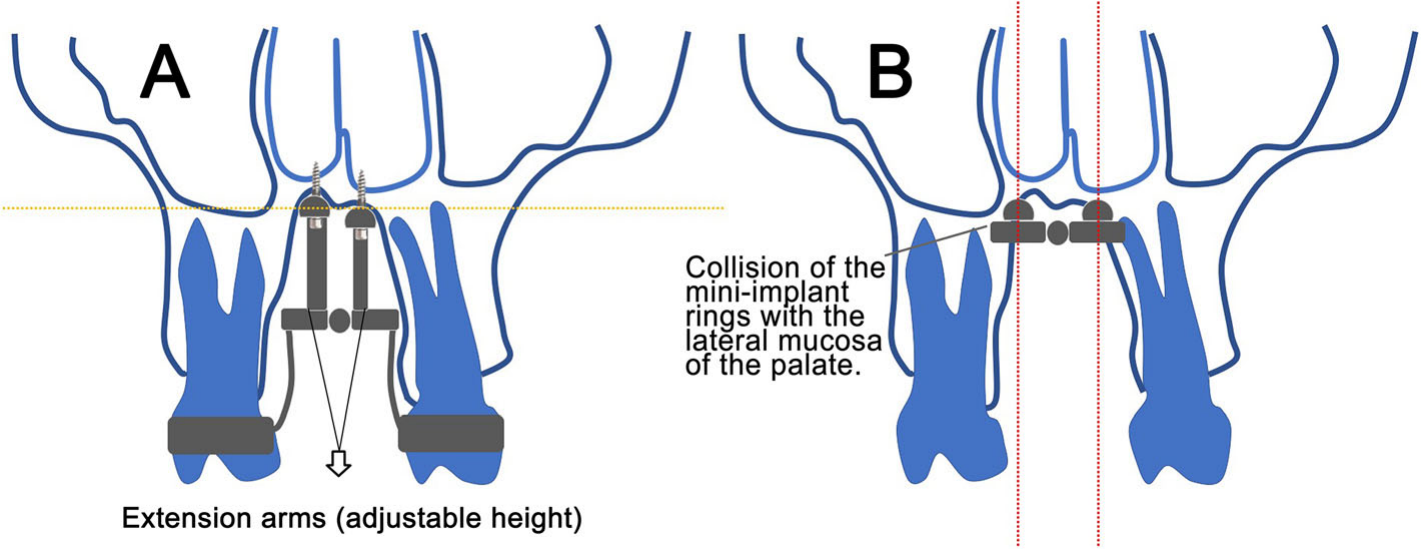
Schematic drawing of a coronal view of a patient with severe transverse maxillary deficiency. **a** The adjustable height of model A arms can provide the RME. **b** Models B and C simulation, the body screw expander collides with the lateral mucosa, which could cause injury

Three different models of MARME, based on three MARME devices (Fig. 2), were digitally created (Fig. 3). Model A comprised five components: one body screw expander and four adjustable arms with rings for mini-implant insertion. These arms have an individualized height adjustment that allows MARME positioning according to the patient's palatal anatomy (Figs. 2a and 3a), thereby preventing body screw expander collision with the lateral mucosa in severe cases of maxillary deficiency (Fig. 4). Model B was a maxillary expander with screw rings joined to the body (Figs. 2b and 3b). Model C was similar to model B, except that model C had open rings for the insertion of the mini-implants (Figs. 2c and 3c), allowing the orthodontist to angle the mini-implants during their insertion. The distance between the anterior and posterior mini-implants was different in each model. The anteroposterior distance in models A, B, and C was 15 mm, 10 mm, and 9 mm, respectively.

The models were created using computer-aided design (Fig. 4) in the software Rhinoceros (Rhinoceros 5.0 SR11 - Robert McNeel & Associates, Seattle, WA). The same process was used to create the mini-implants [17], which were specific to each MARME appliance tested, although all of them were quite similar. A bicortical insertion of the mini-implants was performed, since it showed better RME results as shown by Lee et al. [18]. The MARME complex was positioned in the distal region of the first upper premolar and distal to the first upper permanent molar [13].

The elastic properties of the materials used are listed in Table 2. To apply the FEM, the structures were divided into triangular meshes (elements) whose vertices were the nodes (quantitative data is presented in Table 3). Structural simulation was analyzed using ANSYS software (Mechanical Release 18.2, Canonsburg, PA) of the type “Static and Linear Analysis.”

**Table 2:** Elastic properties of the MARME model material

Material	Modulus of elasticity	Poisson’s ratio
Stainless Steel	200.000	0.33
Titanium	105.000	0.34

**Table 3:** Number of nodes and elements of the MARME models

MARME model	Nodes	Elements
Model A	2.922.005	2.050.160
Model B	1.456.578	984.598
Model C	2.218.113	1.516.929

Images were captured at a maximum principal stress of 7.85 N (1 complete activation screw), according to a previous study [17]. Force distributions were presented in different colors, varying according to the resulting force expressed in each region (Table 4). Results were expressed in MPa (Megapascal) and converted into gf/mm^2^. To make the data easier to interpret, the data were arranged in progressive levels from S1 to S3 according to the degree of tension (Table 5, Fig. 17). This classification was created using the following parameters according to the results observed in the software cited above:

**Table 4:**
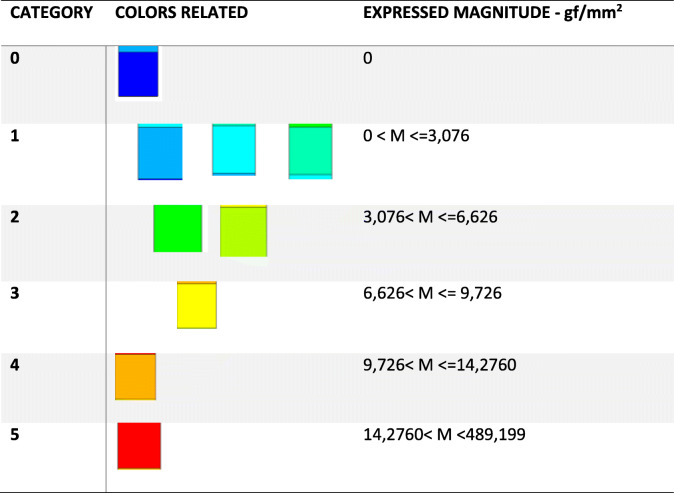
Qualitative classification of the stresses from the 7.85 N (785 g) force magnitude applied

**Table 5:** Categories of stress distribution

Distribution	Category
Low coverage area	S1
Mean coverage area	S2
High coverage area	S3

**Fig. 5:**
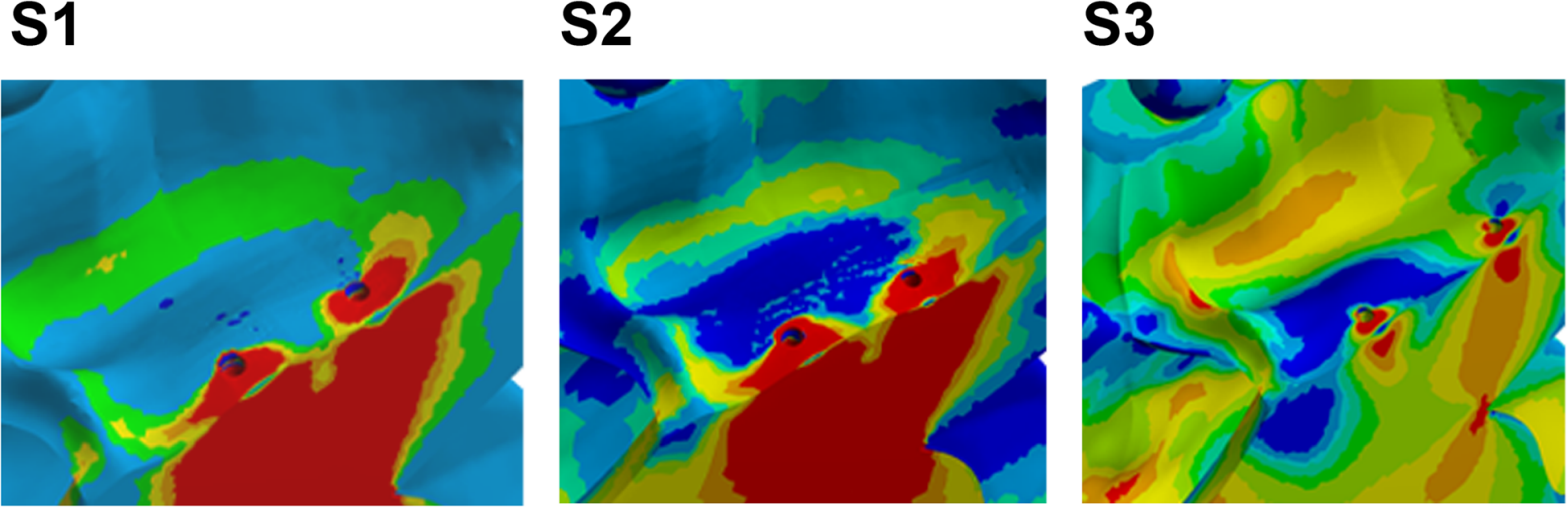
Quantity of distribution per square millimeter. Categories S1 (low coverage area), S2 (mean coverage area), and S3 (high coverage area)

- Color grading (quantity and color nuances)

- Stress range in mm^2^

Smooth color and no graduation (S1) implied that there was only a low intensity, undistributed stress peak, as shown in the green areas in Fig. 4 (S1). On the other hand, Fig. 4 (S2) shows a color gradient from green to blue, which, according to the software used, implied that there was a stress distribution. Figure 4 (S2) still shows a larger area of tension distribution. In contrast, Fig. 4 (S3) shows a color graduation between orange, yellow, shades of green, and blue, which indicates a large distribution of tension, besides having the largest area of tension distribution per mm^2^.

## Results

**Fig. 6:**
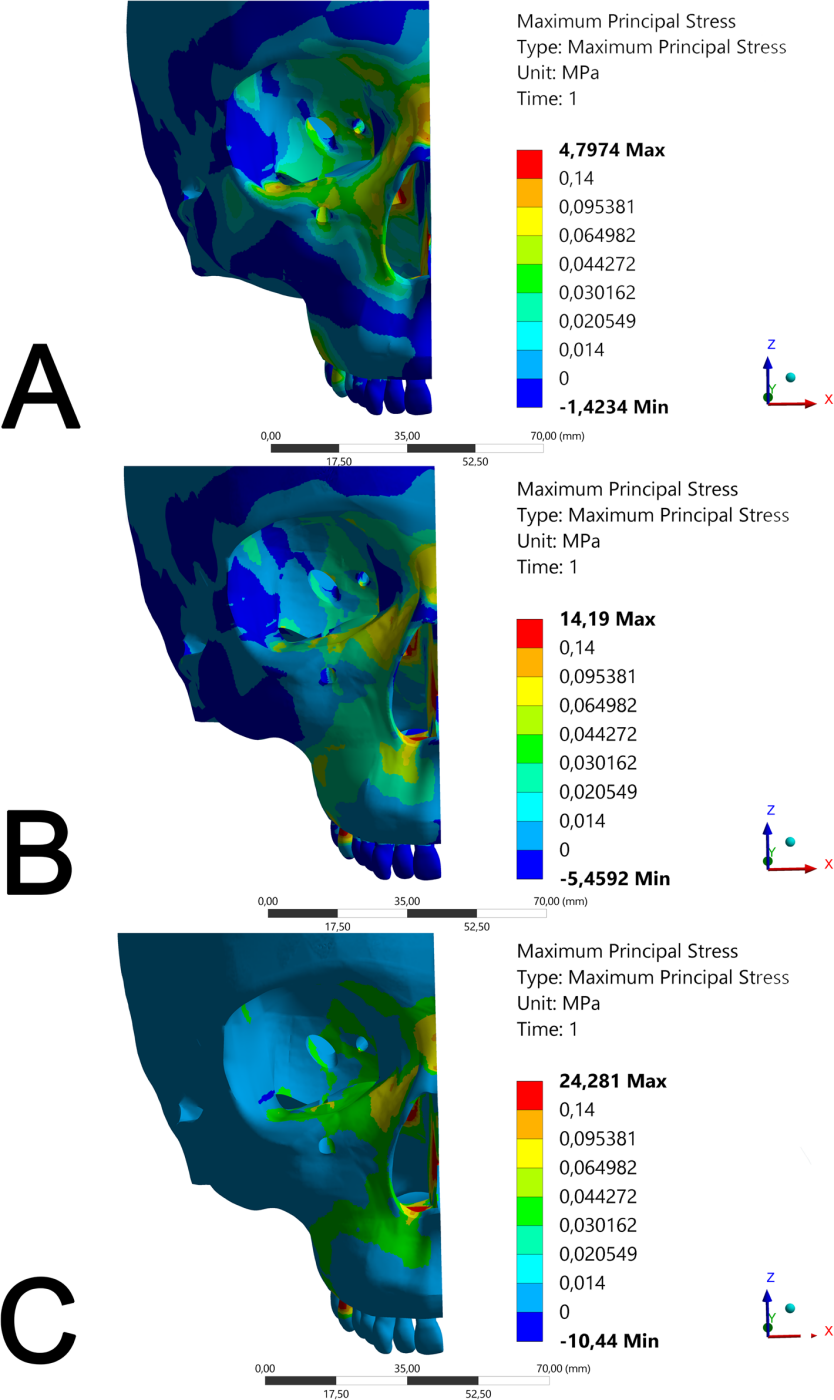
FEM simulation in frontal view

**Fig. 7:**
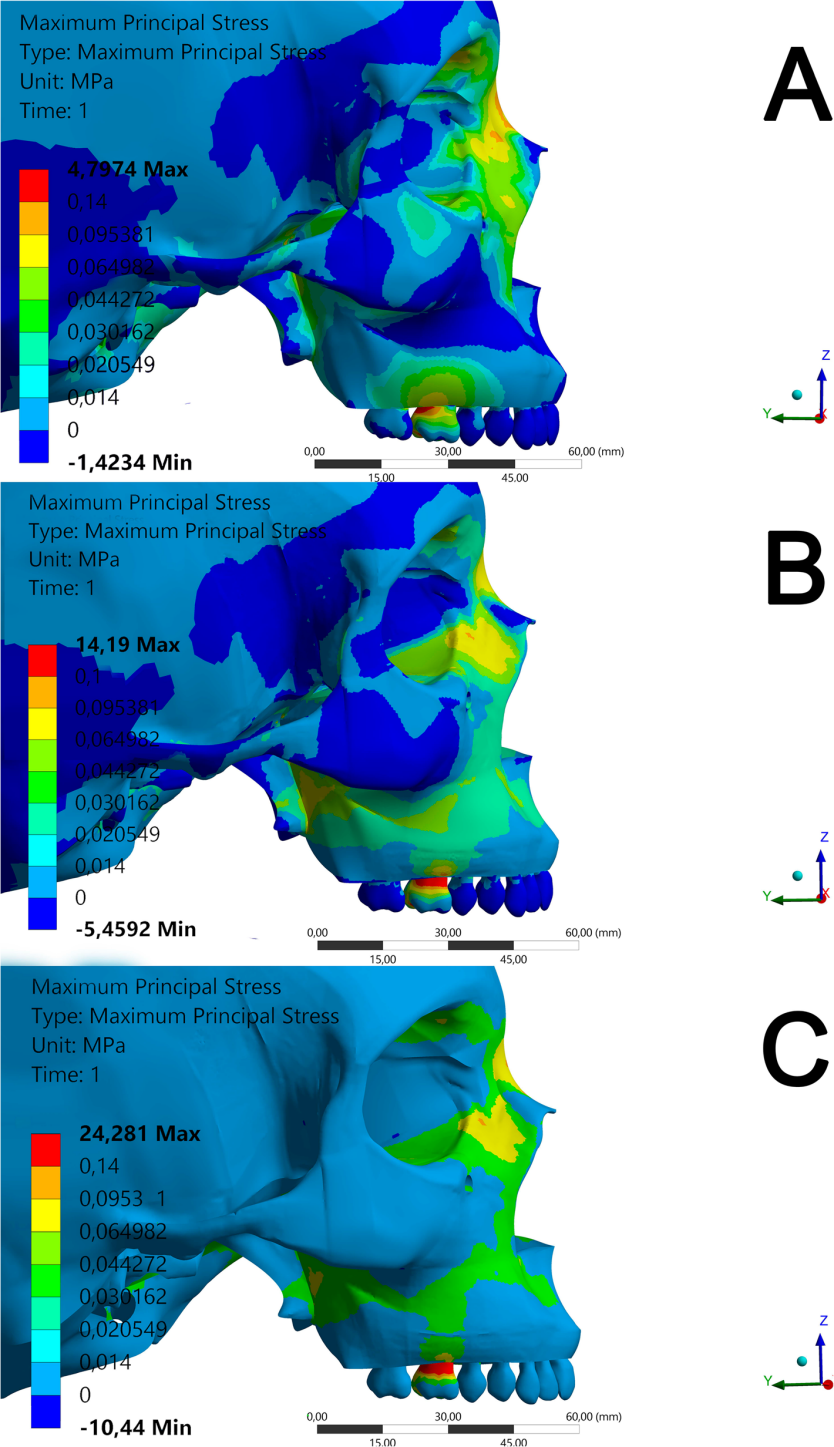
FEM simulation in lateral view

**Fig. 8:**
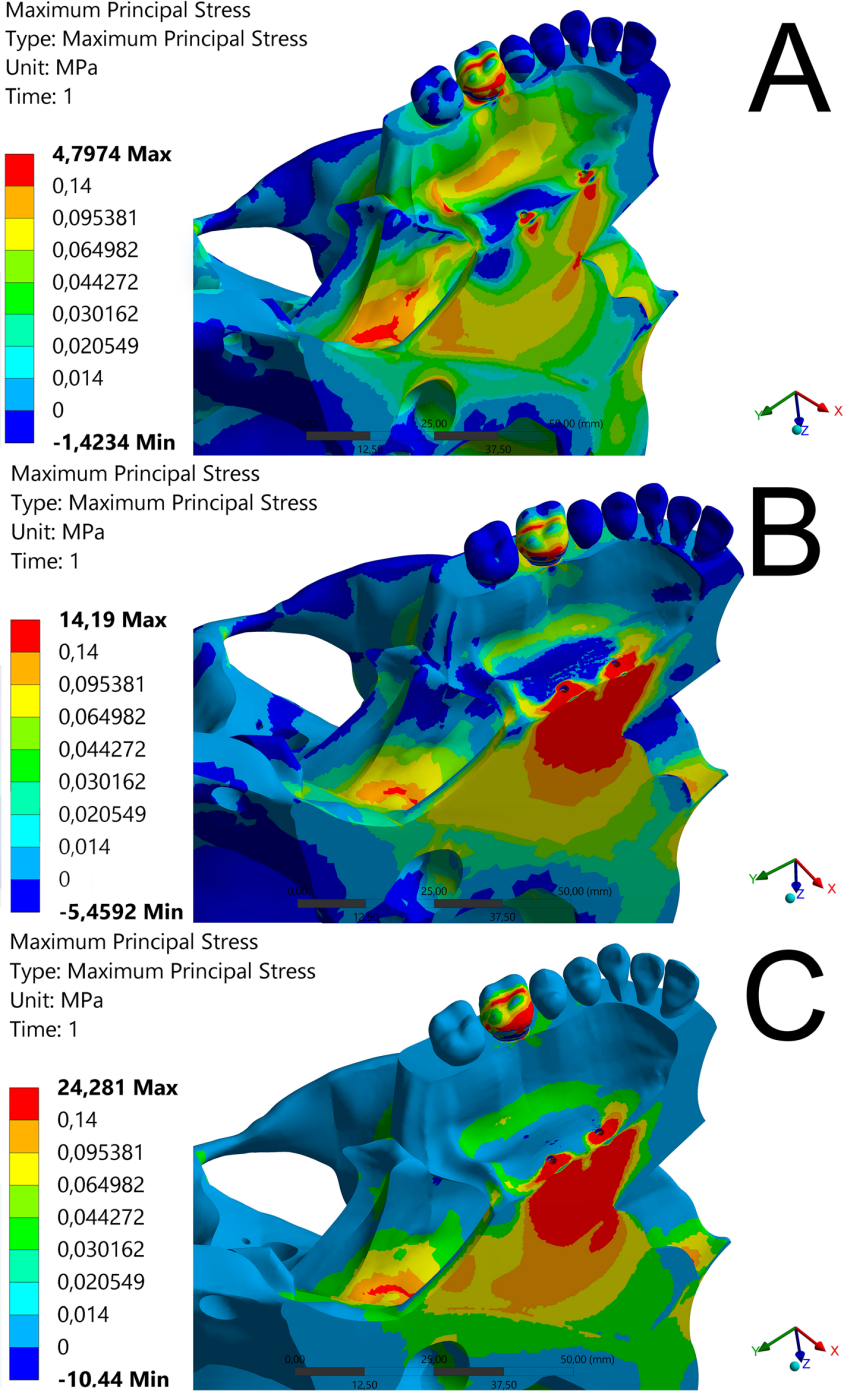
FEM simulation in isometric occlusal view

Results on the stress category and distribution for each view and structure evaluated are presented in Table 6. The different stress regions are represented in Figs. 6, 7 and 8 and Videos 1, 2, 3, 4, 5, 6, 7, 8 and 9.

**Table 6:** Category and distribution results in each evaluated view and structure

MARME model	Model A		Model B		Model C	
View/structure	Category	Distribution	Category	Distribution	Category	Distribution
Frontal/maxillary bone	2 and 3	S3	2 and 3	S2	2	S1
Frontal/nasal region	1 and 4	S3	1 and 3	S2	1 to 3	S1
Frontal /infraorbital region	2 and 3	S3	2 and 3	S2	2	S1
Inclined frontal/infraorbital region	2 and 3	S3	2 and 3	S2	2	S1
Inclined frontal/mini-implants region	1 to 5	S3	1 to 5	S1	1 to 5	S1
Inclined frontal/tooth	1 to 3	S3	3 to 5	S2	3 to 5	S2
Inclined frontal/nasal region	1 and 4	S3	1 and 3	S2	1 to 3	S1
Isometric frontal/maxillary region	0 to 3	S3	2 and 3	S2	2	S1
Isometric frontal/tooth	1 to 4	S3	2 to 5	S2	2 to 5	S2
Isometric frontal/infraorbital region	1 to 3	S3	1 to 3	S2	2 to 3	S1
Isometric frontal/nasal region	1 and 4	S3	1 and 3	S2	1 to 3	S1
Lateral/lateral lamina of pterygoid process	1 to 3	S3	1 to 3	S3	2	S1
Lateral/nasal region	1 and 4	S3	1 and 3	S2	1 to 3	S1
Lateral/tooth	1 to 5	S3	1 to 5	S2	2 to 5	S2
Isometric occlusal/mini-implants region	1 to 5	S3	1 to 5	S2	2 to 5	S1
Isometric occlusal/lateral lamina of pterygoid process	1 to 3	S3	1	S1	0	-
Isometric occlusal/tooth	1 to 5	S2	1 to 5	S3	2 to 5	S1
Vestibular/lateral lamina of pterygoid process	1 to 3	S3	1 to 3	S3	1 to 2	S1
Vestibular/tooth	1 to 5	S3	1 to 5	S2	2 to 5	S1
Occlusal/lateral lamina of pterygoid process	1 to 3	S3	1 to 3	S2	2	S1
Occlusal/mini-implants region	1 to 5	S3	2 to 5	S2	2 to 5	S1
Occlusal/tooth	1 to 5	S2	1 to 5	S3	1 to 5	S1
Occlusal/junction of the vomer wing with the medial lamina of the sphenoid process region	1 to 5	S3	1 to 5	S3	1 to 5	S3

The categories are presented according to Table 4 and distribution according to Table 5

In the frontal view (Fig. 6), high stress points occurred in the mini-implant region and in the supporting tooth region (upper first molar) for models B and C (Fig. 6b, c, respectively). For model C, the stress force was of category 3, which means approximately 9.726 gf/mm^2^. Model B showed the same amount of force with a wider distribution area. For model A (Fig. 6a), a better distribution of these tensions, as well as a higher force intensity ranging in category 4 with up to 14.2760 gf/mm^2^ in the nasal region, were observed. Tensions in models B and C were concentrated on the buccal bone plate (Figs. 6b and 5c). In general, there were tensions in orbit (all models presented a maximum principal stress of 6626 gf/mm^2^) and the distribution was higher in model A, followed model B and C (Fig. 6). The strongest stress point, category 5, occurred in the nasal cavity floor, reaching up to 489.199 gf/mm^2^ at the insertion region of the mini-implants, for both models B and C. It was also observed that tension was lower on teeth for model A (9.726 gf/mm^2^), while both models B and C reached category 5 with up to 489.199 gf/mm^2^.

The stresses in the nasal region were similar in the frontal view for all three models. In the frontal isometric view, the vestibular stress region for models B and C was evident, ranging from 6.6263 to 9.7262 gf/mm^2^, while the force distribution for model A varied from 3.0757 to 9.7262 gf/mm^2^, with better stress distribution. The stress on the tooth region for model A was of category 4 (maximum principal stress of 14.2760 gf/mm^2^), and the other models reached category 5 with stress value of 489.199 gf/mm^2^. The nasal region presented tensions up to 14.2760 gf/mm^2^ for model A with force distribution category S3.

In the lateral view (Fig. 7; Videos 1, 2 and 3), the stress on the lateral lamina of the pterygoid process of the sphenoid bone was evident. Model C (Fig. 8c; Video 3) presented a stress peak up to 6.626 gf/mm^2^ (category 2) only in the central region of the lateral lamina of the pterygoid process. Model B (Fig. 6b; Video 2) presented a well-distributed stress ranging from 6.626 to 9.726 gf/mm^2^, featuring categories 2 and 3. Model A (Fig. 7a) presented the same stress range between 6.6263 and 9.7262 gf/mm^2^ as observed in model B, and the distribution was transmitted through the entire pterygoid process (Video 1). The nasal region also showed stress tensions up to 14.2760 gf/mm^2^ on model A, with force distribution category S3. Interestingly, the tooth tension was lower in this view (Fig. 7).

In the vestibular view (Videos 4, 5 and 6), tensions in the lateral lamina of the pterygoid process presented a very distinct force distribution. For model C (Video 6), the peak tension was up to 6.626 gf/mm^2^ (category 2). Models A and B (Video 4 and 6) presented with category 3 (9.726 gf/mm^2^), in a wider area and maximum tension. For model A, in the lateral lamina of the pterygoid process (Video 4), the tension showed great distribution reaching up to 9.726 gf/mm^2^. In the vestibular view, all models reached a maximum stress of 489.199 gf/mm^2^ in the supporting tooth, and model A obtained the most uniform distribution (S3).

In the isometric occlusal view (Fig. 8; Video 7, 8, 9), a large area of high-intensity tension of up to 489.199 gf/mm^2^ (category 5) was observed at the site of installation of the mini-implants in models B and C (Fig. 7b, c). Even though model A (Fig. 8a) presented similar amount of tension, it was distributed all over the palate, up to the lateral lamina of the pterygoid process (Video 7). Model C (Fig. 8c) did not present any tension in this area (Video 9), and model B presented tension of only 3.076 gf/mm^2^ (category 1) at few points (Fig. 8b; Video 8). All tested models reached a maximum stress of 489.199 gf/mm^2^ in the tooth region, where model B obtained the best force distribution (S3).

Occlusal view clearly showed tension distribution from the maxillary bone to the lateral lamina of the pterygoid process. For model A, the tension was up to 9.726 gf/mm^2^ (category 3). In this view, a high stress area (category 5) was observed in the nasal floor cavity at the insertion point of the mini-implants. In model A, the distribution appeared with a lower stress area and wider distribution of forces across the palate (Table 6). Model B showed lower molar stress in the occlusal view.

## Discussion

FEM methodology was chosen for this study because it is a precise mathematical process, which could test the mechanical quality of three MARME models. These devices were chosen because of their different advantages. Model A was designed to perform RME in cases of severe atresia [11]. Model B was chosen because it has already shown satisfactory results in clinical studies [19, 20, 21]. Model C was chosen because it has open rings for insertion of mini-implants, which is advantageous in situations of mini-implant loss (it is possible to reposition another mini-implant with a different angulation from the original installation) [10, 22].

Regarding the stress distribution found in the supporting tooth region, high stress intensity up to 489,199 gf/mm2 was found in general (Fig. 5, Table 6). In the lateral (Fig. 6a, Video 1) and vestibular views (Video 4), the maximum stress region observed in model A was in the distal portion of the tooth that received the band. Models B and C (Fig. 6c, b) presented extremely high forces (category 5—maximum principal stress 489.199 gf/mm^2^) in the mesial, buccal, and distal faces. Jain et al. 2017 [17] concluded that excessive force on the supporting teeth is closely related to the side effect of tooth tipping. Clinically, a high incidence of tooth tipping (90.1%) [23] after RME was reported with a model similar to that of model B [23, 24]. Another clinical study found increased tooth inclination in the right and left upper molars of 2.77° and 2.03°, respectively [9]. Hence, it could conceivably be hypothesized that model A has a minor side effect of tipping of teeth. However, as the periodontal tissue was not included in the evaluation, the amount of stress on the supporting teeth cannot suggest reliable clinical implications.

All evaluated models presented tensions in the infraorbital region. Model C (Figs. 5c and 6c) presented the lowest distribution of these stresses (6626 gf/mm^2^), while model B (Figs. 5b and 6b) presented low and medium stress (ranging from 3.076 to 9726 gf/mm^2^) and a relatively non-diffused distribution. Model A presented well-distributed tensions (S3), varying from low to medium stress intensities (from 3.076 to 9726 gf/mm^2^—Figs. 5a and 6a). This corroborates previous studies on MARME [16, 21]. As it is a region with important nerves, more clinical studies are necessary to assess whether there are any side effects in this region.

Models B (maximum principal stress of 6626) and C (from 6.626 to 9.726 gf/mm^2^) showed an external stress distribution pattern (Fig. 5b, c), corresponding to the buccal alveolar bone surface, with a better stress distribution in model B (S2). Reportedly, RME conventional treatment can change buccal bone thickness [7, 7]. This corroborates with a previously published FEM study of MARME [9]. In a clinical study, Moon et al. [9] reported a reduction in the buccal cortical thickness of the alveolar bone of 0.67 ± 0.44 mm on the right upper molars and 0.48 ± 0.48 mm on the left upper molars. Similar results were demonstrated by Lim et al. [23]. Furthermore, Ngan et al. [27] observed a 39% reduction in buccal cortical thickness after the use of a model similar to model B in their clinical study. In the present study, model A presented more internal tension in the maxillary bones and an S3 distribution in the buccal region (Fig. 26a; Video 27). This distribution included the internal portion of the bone, but greater intensity of stress was observed in the nasal bone and lateral lamina of the pterygoid process. Future clinical studies are necessary to evaluate the clinical consequences of these results.

Nasal, frontonasal, and internal sutures received tensions of up to 9.726 gf/mm^2^ in models B and C (Fig. 5b, c). Similar results were observed previously with the same magnitude of transverse force application [17]. Model B showed a better force distribution (Table 6). Model C showed the same stress levels as model B but showed a low distribution of stress. Model A showed up to 14.2760 gf/mm^2^ of stress with S3 distribution category. Song et al. [28] observed similar results in their clinical study and showed that frontonasal and frontomaxillary sutures underwent major changes after RME, which were more desirable than the changes found in the sutures involved with the zygomatic bone. Furthermore, another clinical study showed a significant increase in the cross-sectional dimension of the nasal cavity (in the premolar and molar region) and a consequent improvement in nasal respiratory flow [29]. Therefore, changes in this region are desirable. It is speculated that model A has better effects on the patient's respiratory flow. Longitudinal clinical studies will be necessary to evaluate these changes.

Considering the three models in occlusal view, the region of the medial lamina of the sphenoid process with the wing of the vomer was the location where the models were similar, both in maximum stress and distribution (489,199 gf/mm^2^). In a previous prospective clinical study, changes were observed in the sphenoid process with the use of a model similar to model B [28]. According to the author, more clinical studies should evaluate RME alterations in this region because of the presence of important vessels and nerves.

The expander bodies of the three models have different sagittal distances between the mini-implants. The higher distance of model A allows a position with a greater amount of bone thickness in the anterior region while keeping the posterior mini-implants in a more posterior position (higher resistance region during RME) [30]. Model A fits into a larger portion of the maxilla, which suggests better stress distribution in the palate and the region of the mini-implants.

MacGinnis et al. [16] found high stress around the mini-implants of a model similar to model B, without a wide palatal distribution. These findings are alarming because high-intensity forces with no stress distribution can bend or fracture the mini-implants. Additionally, with this weak tension distribution, the opening of the midpalatal suture may not occur [31].

Lack of tension or distribution in the lateral lamina of the pterygoid process area may result in the failure of RME [30]. The images suggest that model A had a better effect on this area, both in amount of force and quality of the distribution, when compared to model B and even greater effect, when compared to that of model C. Model A showed the best stress distribution pattern in the maxillary bone, nasal region, as well as in the mini-implant insertion region.

Another interesting advantage of model A is that, owing to the height adjustment of the rings, it is possible to place the body screw expander with wider screw sizes. In this way, treatment possibilities are extended with the use of this device.

MARME in adults is a relevant and current topic for orthodontics. Previous evidence already shows that this is a promising method, which should be accurately indicated. Thus, more clinical research is needed to clarify the influence of differences in installation sites, distance from the expander body to the palate, appliance design, and activation protocols.

## Limitations

The limitation of the present study was not to include the periodontal tissue and the muscles in the FEM evaluation.

## Conclusions

Model A presented the best conditions of skeletal stress distribution in a hemi-skull model.

The palate-to-appliance distance, and the increased distance between the mini-implants (characteristic of model A), seem to be the factors that provide greater amplitude of stress distribution in the craniofacial structure.

In cases of severe atresia, model A seems to be an excellent option, since it demonstrated a better distribution of forces along the craniofacial structures.

Clinical studies are necessary to understand and verify the effectiveness of model A.

## Supplementary Information

**Additional file 1.**

**Additional file 2.**

**Additional file 3.**

**Additional file 4.**

**Additional file 5.**

**Additional file 6.**

**Additional file 7.**

**Additional file 8.**

**Additional file 9.**

## Data Availability

The datasets used and/or analyzed during the current study are available from the corresponding author on reasonable request.

## Abbreviations

RME: Rapid maxillary expansion
SARME: Surgically assisted rapid maxillary expansion
MARME: Mini-implant assisted rapid maxillary expansion
FEM: Finite element method
DICOM: Digital imaging and communication in medicine
